# The origin and role of innate lymphoid cells in the lung

**DOI:** 10.1186/s40779-016-0093-2

**Published:** 2016-08-19

**Authors:** Deng-Ming Lai, Qiang Shu, Jie Fan

**Affiliations:** 1 Department of Cardiovascular Surgery, the Children’s Hospital of Zhejiang University School of Medicine, Hangzhou, Zhejiang 310052 China; 2 Department of Surgery, University of Pittsburgh School of Medicine, Pittsburgh, PA 15213 USA; 3 Research and Development, Veterans Affairs Pittsburgh Healthcare System, Pittsburgh, PA 15240 USA

**Keywords:** Innate lymphoid cells, Innate immunity, Lung diseases, Airway, Cell interaction, Cytokines

## Abstract

Innate lymphoid cells (ILCs), a newly identified member of the lymphoid population, play a critical role in the transition from innate to adaptive immunity in host defense. ILCs are important in mucosal barrier immunity, tissue homeostasis, and immune regulation throughout the body. Significant alterations in ILC responses in lung diseases have been observed and reported. Emerging evidence has shown that ILCs are importantly involved in the pathogenesis and development of a variety of lung diseases, i.e., helminth infections, allergic airway inflammation, and airway hyper-responsiveness. However, as a tissue-resident cell population, the role of ILCs in the lung remains poorly characterized. In this review, we discuss the role of ILCs in lung diseases, the mechanisms underlying the ILC-mediated regulation of immunity, and the therapeutic potential of modulating ILC responses.

## Background

**Fig. 1:**
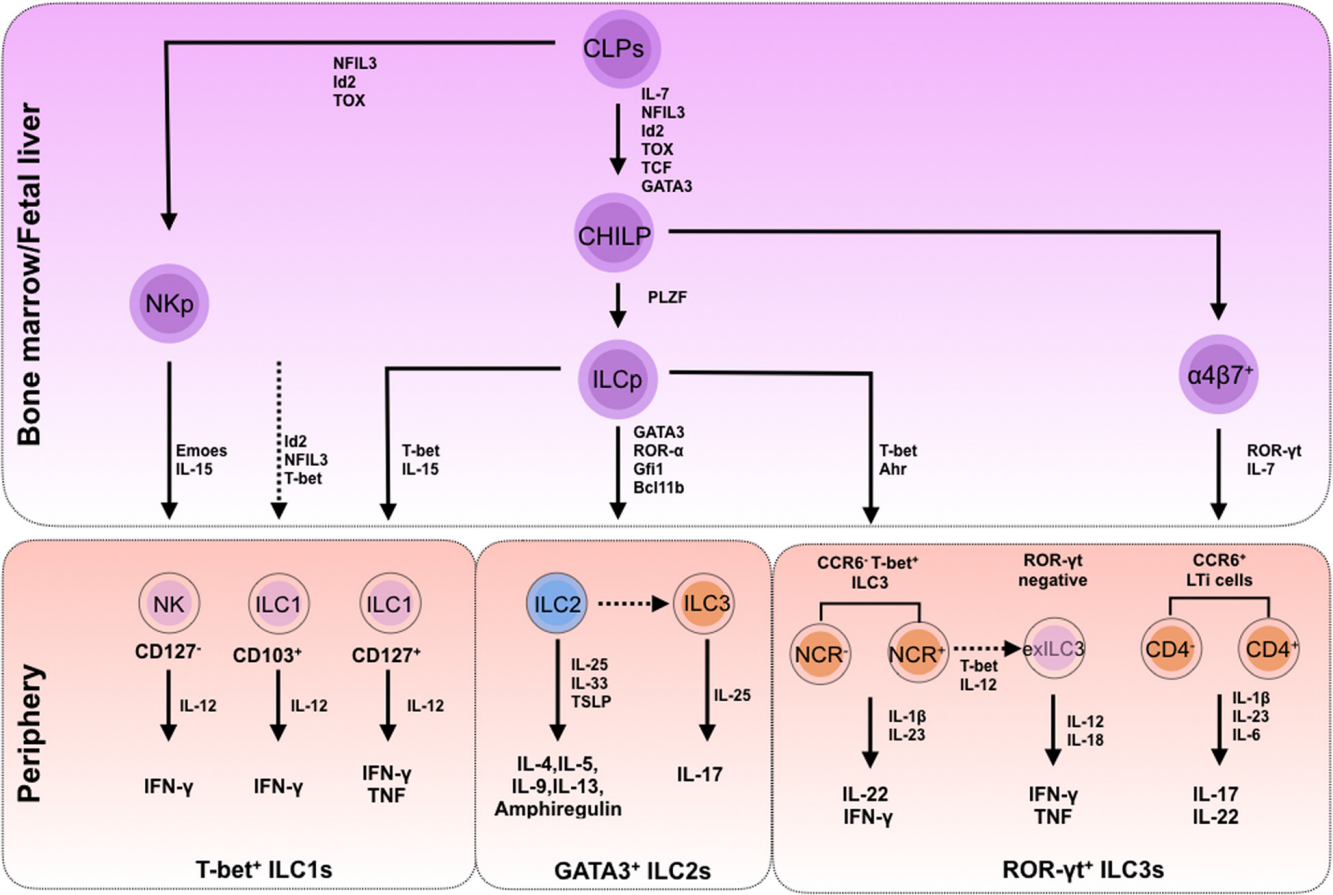
Development and heterogeneity of the ILC family. ILCs develop from distinct progenitors in the fetal liver or bone marrow and then develop into mature ILCs in the periphery. Different transcription factors and cytokines are involved in the development of the three groups of mature ILCs. All ILCs develop from CLPs, which can differentiate into NKps or CHILPs. CHILPs can further differentiate into LTi cells through α4β7+ populations or into other ILC populations through differentiation into ILCps. ILC1s express T-bet, are responsive to IL-12, and produce IFN-γ and/or TNF. ILC2s highly express GATA3, are responsive to IL-25, IL-33 and TSLP, and produce IL-4, IL-5, IL-9, IL-13 and amphiregulin. ILC3s express ROR-γt, are responsive to IL-1β and IL-23, and produce IL-17 and/or IL-22

Innate lymphoid cells (ILCs) are emerging as an important cell population of innate immunity and play complex roles in lymphoid tissue formation, tissue remodeling, tissue stromal cell homeostasis, and regulation of host responses to infection and inflammation. Compared to adaptive lymphocytes, ILCs are relatively rare in lymphoid tissue, but they populate barrier surfaces, such as the skin, intestine, and lung, as well as in adipose and some mucosal-associated lymphoid tissues [1–3]. Compared to typical lymphoid cells, ILCs are characterized by three main features: 1) the absence of recombination activating gene (RAG)-dependent rearrangement of antigen receptors; 2) a lack of phenotypical markers of myeloid cells and dendritic cells; and 3) particular lymphoid morphology [4]. The prototypes of the ILCs family are natural killer (NK) cells and lymphoid tissue-inducer (LTi) cells, which were discovered in 1975 and 1997, respectively [5, 6]. Recently, other members of the ILC family have been characterized. Based on their phenotypical and functional characteristics, ILCs are categorized into three subgroups. ILC1s include NK cells, which produce interferon-γ (IFN-γ). ILC2s produce type 2 cytokines, e.g. IL-5 and IL-13, and are dependent on GATA-binding protein 3 (GATA3) and retinoic acid receptor-related orphan receptor-α (ROR-α) for their development and function. ILC3s include all ILC subtypes that produce IL-17 and/or IL-22, and they depend on the transcription factor ROR-γt for their development and function (Fig. 1) [2, 7].

The molecular and cellular components of the innate and adaptive immune systems influence and regulate both lung homeostasis and the development of lung diseases. The emerging ILC family has been shown to have critical roles in the initiation, modulation, and resolution of lung diseases. Studies using mouse models indicated that ILCs play an important role in the remodeling of damaged lung tissue following influenza virus infection, contribute to the exacerbated allergic asthma induced by viruses [8–10], promote the release of inflammatory mediators in allergic lung inflammation [11] and are involved in the induction of pulmonary fibrosis [12]. Human studies showed that ILC responses in pulmonary diseases are substantially altered. These findings suggest broad roles of ILCs in lung physiological and pathological process. In this review, we address the origin, development, and heterogeneity of ILCs, and the roles of ILCs in lung homeostasis and diseases; we will then discuss potential novel strategies of intervention in lung diseases by targeting ILCs.

### Development and heterogeneity of the ILC family

Common lymphoid progenitors (CLPs) differentiate into cells of the adaptive immune system, i.e., T cells and B cells; however, a subset of CLPs gives rise to ILCs [13, 14]. ILCs initially develop in the fetal liver and later develop in the adult bone marrow [14–16]. The development of ILCs from CLPs is independent of the RAG-dependent rearrangement of antigen receptors, but is regulated by several transcription factors [3, 17, 18]. The differentiation of ILCs from CLPs requires transcriptional factors, including inhibitor of DNA binding 2 (Id2), nuclear factor interleukin-3 regulated (NFIL3), and thymocyte selection-associated high-mobility group box protein (TOX) [2, 3, 16, 19–24]. Firstly, two precursors, including NK cell precursors (NKps) and common helper innate lymphoid precursors (CHILPs), are derived from CLPs. NKps develop into NK cells, and CHILPs give rise to all other ILCs through a process that requires T-cell factor 1 (TCF1), interleukin 7 (IL-7) [24] and GATA3 [25–28]. From CHILPs, several distinct progenitors expressing α4β7 integrin develop into LTi cells [14]. Another group of CHILPs can give rise to ILC progenitors (ILCps) [15]. The development of ILCps from CHILP depends on promyelocytic leukemia zinc finger (PLZF), a transcriptional repressor involved in the cell cycle control, development and differentiation of myeloid cells [15]. ILCps transiently express abundant PLZF, a transcription factor associated with NKT cell development [29, 30], suggesting an interaction between ILCps and NKT cells. Studies have identified a population of human CD34^+^ hematopoietic progenitor cells (HPCs) in human bone marrow and peripheral blood that express ROR-γt and selectively differentiate toward ILC3s. HPCs are located in the tonsils and intestinal lamina propria (LP) and are able to differentiate into either NK cells or LTi-like cells through a process influenced by aryl hydrocarbon receptor (Ahr) ligands, stem cell factor (SCF), and IL-15 [31]. Under the regulation of different transcription factors and interleukins, NKps, ILCps, and α4β7^+^ progenitors differentiate into three groups of ILCs, termed Group 1 ILCs (ILC1s), Group 2 ILCs (ILC2s) and Group 3 ILCs (ILC3s) (Fig. 1) [1–3, 7].

ILC1s respond to IL-12, constitutively express T-bet, and produce effector cytokines, such as interferon (IFN)-γ and tumor necrosis factor (TNF) [16, 32, 33]. The differentiation of ILC1s from ILCps requires eomesodermin (Eomes), T-bet, IL-15, and IL-7. Based on the difference in the expression of the products and requirements for the regulators, ILC1s can be divided into at least three subsets. One subset includes conventional NK cells, which require Eomes and IL-15, but not IL-7 or T-bet, for development from NKps [16]. Another subset of ILC1s, CD103^+^ intraepithelial ILC1s, develop from an as yet unknown precursor in a process that requires T-bet, NFIL3, and Id2, but not IL-15; ILC1s express Eomes [33]. Further studies also identified a subset that develops from CHILPs and ILCps in a T-bet- and IL-15-dependent, but IL-7-independent, manner and does not express Eomes (Fig. 1) [16, 32].

ILC2s respond to the cytokines IL-25, IL-33, and thymic stromal lymphopoietin (TSLP); they constitutively express high levels of GATA3; and they produce the effector cytokines IL-4, IL-5, IL-9, IL-13, and amphiregulin [8, 34, 35]. ILC2 development requires GATA3 [27, 28], ROR-α [36, 37], and the transcription factors Gfi1 and Bcl11b [38–40]. Furthermore, GATA3 is required for the functional maturation and maintenance of ILC2s [41–44], and Bcl11b serves as a upstream regulator of the transcriptional factor Gfi1 to maintain Gfi1 expression in mature ILC2s. Mature ILC2s populate the healthy skin, lungs, and adipose tissue of humans and mice [8, 34, 35, 45–48], and they are the most common group in the lung [49].

ILC3s respond to IL-1β, IL-6, and IL-23; they constitutively express ROR-γt, and they produce the effector cytokines IL-17 and/or IL-22 [50–53]. LTi cells are members of the ILC3s, which develop independently of PLZF-dependent ILCps. They express CCR6 and/or CD4 and secrete IL-22 and IL-17 and lymphotoxin (LT) [54]. A subset of adult ILC3s develop from ILCps and can be divided into two groups, NCR^-^ ILC3s and NCR^+^ ILC3s, according to the expression of natural cytotoxicity receptors (NCRs, e.g., NKp46 and NKp44). These cells can express IFN-γ and IL-22, and their development requires T-bet and Ahr [54–58]. T-bet^+^ ILC3s are nearly exclusively located in the skin and intestinal lamina propria, whereas LTi-like ILC3s are positioned in the intestine and lymphoid tissues. However, ILC3s with a unique phenotype, which express Thy1, stem cell antigen 1 (SCA-1), ROR-γt and IL-23R, can develop in some inflammatory contexts in the liver and intestine [59, 60].

Inter-transfer between different types of ILCs has been reported. The ILC2 population can differentiate into IL-17-producing ILC3-like cells [61]. Many factors are involved in the functional transfer between ILC2s and ILC3s. Bcl11b seems to be a determinant for the maintenance of the ILC2 phenotype because Bcl11b-/- ILC2s lose their ILC2 functions and gain ILC3s functions. Interestingly, in response to the protease allergen papain, these cells expand and produce ILC3, but not ILC2, cytokines and cause increased airway infiltration of neutrophils rather than eosinophils. Bcl11b has a direct role in repressing the expression of the gene encoding the ILC3s transcription factor Ahr, which is an ILC3 lineage transcription factor, thus contributing to the silencing of ILC3 genes in ILC2s. In Bcl11b^-/-^ mice, the ILC2s down-regulated ILC2s genes, such as Gata3 and Il1rl1 (encoding IL-33 receptor), and up-regulated Rorc and the ILC3 genes. Recent studies reported plasticity between different ILC groups. ILC3 subsets could down-regulate ROR-γt expression in humans and mice, leading to dominant T-bet expression and sustained IFN-γ expression, both of which are known characteristics of ILC1s [32, 54, 55]. These transient cells were termed ex-ILC3s [16, 32, 54, 55]. It is currently uncertain how to define and classify these populations.

### ILCs in the lungs

ILCs in both lymphoid and non-lymphoid organs are tissue-resident cells that are locally renewed and expanded in response to acute environmental challenges. The maintenance of ILCs depends on their self-renewal ability in different microenvironments and physiological conditions [49].

ILCs have been identified in the human and mouse lungs and airways [8]. In the mouse lung, ILC2s are the main cell type of ILCs. However, ILC2s are a relatively rare population, comprising 2-3 × 10^4^ cells in the lung of a naïve mouse and representing 0.4-1 % of the total live cells in the lung [8]. In human lung tissue, approximately 60 % of ILCs are ILC3s, and the most abundant ILC3s are NCR^-^ ILC3s. In the contrast, the percentages of ILC1s and ILC2s are 10 % and 30 %, respectively [62]. ILC2s, although small in number, play important roles in innate immunity and disease progressions by secreting type 2 cytokines and tissue growth factors. Furthermore, in humans, LC3-like cells are observed in the bronchoalveolar lavage fluid of individuals with asthma [63].

Studies have shown that more than 95 % of the lung-resident ILCs are of host origin and are maintained and expanded locally under physiologic or pathological conditions [49]. During lung infections, the local expansion of resident ILCs is followed by the increased hematogenous recruitment and redistribution of ILCs.

### ILCs maintain lung homeostasis

**Fig. 2:**
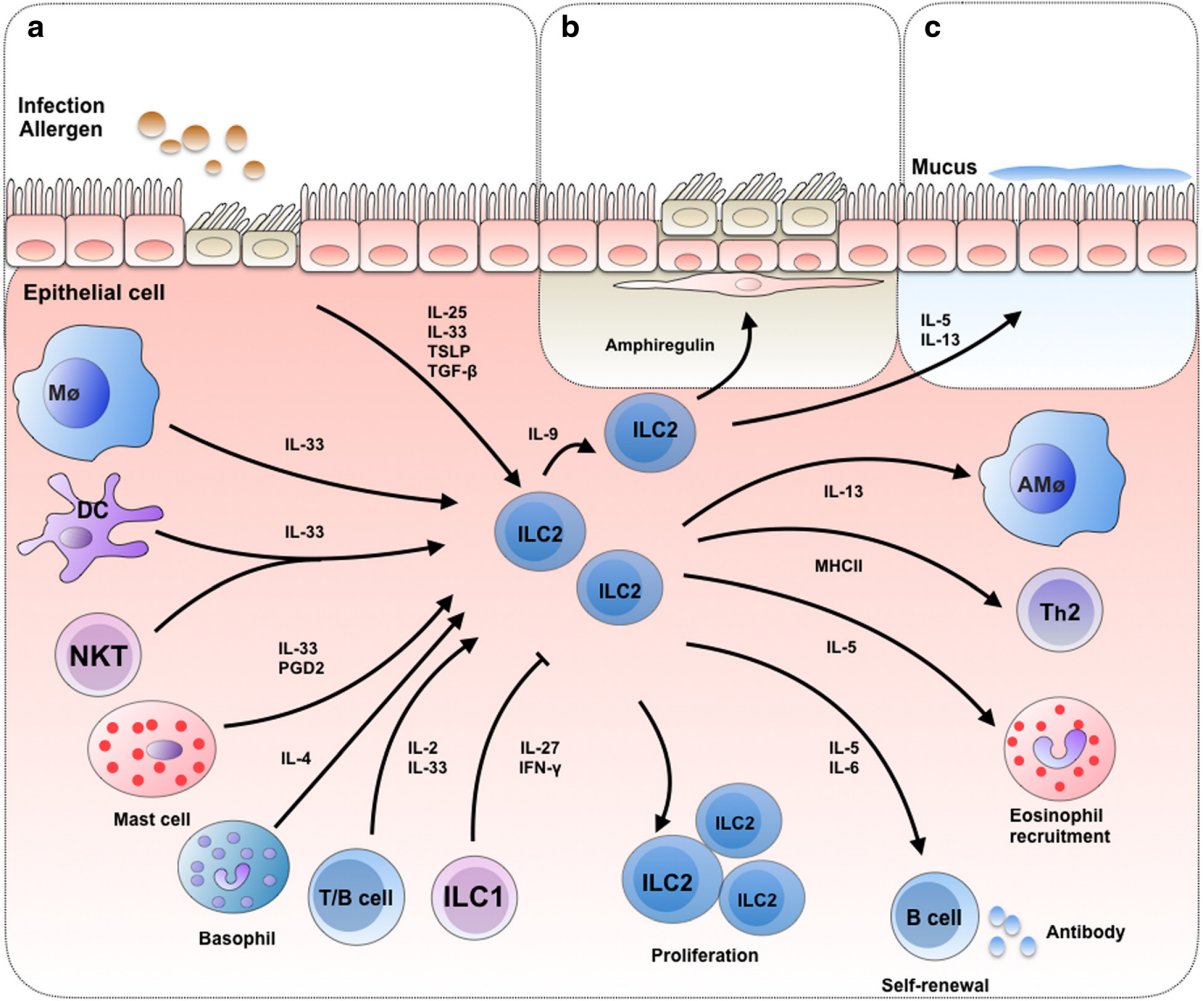
The role of the ILC family in lung homeostasis, cell-cell interactions and tissue repair. **a** In response to infection or allergen exposure, epithelial cell- and myeloid cell-derived IL-25, IL-33 and TSLP elicit ILC2s responses in the lung. ILC2 responses can be enhanced by basophil-derived IL-4 or mast cell-derived PGD2. Activated ILC2s can subsequently promote IL-5-mediated eosinophil recruitment, IL-13-mediated AMac differentiation, or MHCII-mediated enhancement of Th2 cell responses, resulting in allergy and fibrosis. However, ILC1-derived IL-27 and IFN-γ can antagonize the function of ILC2s and type 2 innate immune responses. Furthermore, ILC2s proliferate in response to lymphoid-derived IL-2 and produce large amounts of Th2 cytokines, including IL-5, IL-6 and IL-13. IL-5 and IL-6 regulate B cell antibody production and the self-renewal of B1 cells. **b** After infection in the lung, airway epithelial cells are damaged and produce IL-33. ILC2s respond to IL-33 and produce amphiregulin, which promotes the repair of the airway epithelium. Together with autocrine IL-9 production, the IL-33 produced by macrophages, DCs, mast cells, NKTs and lymphoid cells enhances the repair function of ILCs. **c** In the innate inflammatory response in the lung, alveolar type II cells produce IL-33 and TSLP, which synergistically induce ILC2s to produce IL-5 and IL-13. IL-5 and IL-13 are known to promote mucus production

Maintenance of the epithelial barrier function in respiratory tract mucosal sites is critical to limit exposure to physiological and immunological stimuli [64, 65]. Recent findings suggest that ILCs are a crucial cell lineage for maintaining airway barrier integrity and tissue homeostasis after influenza virus infection [8]. ILC2-induced mediators including IL-4, IL-5, IL-9, IL-13 and amphiregulin are important for maintaining lung homeostasis (Fig. 2) [8, 66]. Mouse and human studies have shown that lung-resident ILC2s are observed in the bronchoalveolar space and can thus be collected from the bronchoalveolar lavage fluid. ILC2 depletion impairs epithelial integrity and lung function following influenza virus infection. Studies further showed that ILC2 depletion resulted in an impaired ability to generate hyper-plastic epithelial cells and led to substantial epithelial degeneration and necrosis [8]. IL-22, which is mainly produced by ILC3s in the lung, is also involved in the maintenance of lung epithelial cell function and in the negative regulation of lung inflammation [67, 68]. A recent study suggested that during lung homeostasis, ILC2s use the IL-9 module to coordinate epithelial cell maintenance [69]. In the chitin-induced innate inflammatory response of the lung, alveolar type II cells produce TSLP and IL-33, which synergistically induce an interferon regulatory factor 4 (IRF4)-IL-9 program in ILC2s. Additionally, autocrine IL-9 promotes the production of IL-5 and IL-13, which are required for optimal epithelial responses in the conducting airways [69]. Moreover, IL-5 and IL-13 were known to promote mucus production and tissue repair [66, 70]. Thus, ILC2s contribute to barrier surveillance and epithelial responses.

### Interaction of ILCs with other cells in the lung

In the lung, ILCs interact with other cells, including local epithelial cells, NKT cells, myeloid cells and granulocytes, to form an immune system network.

Studies have suggested that epithelial cell-derived mediators are important for ILC regulation. For example, epithelial cell- or myeloid cell-derived IL-25, IL-33, and TSLP can elicit ILC2 responses after allergen, chemical, or helminth parasite exposure or influenza virus infection [8, 9, 34, 35, 46, 71–73]. ILC2 responses in the lung can be enhanced by basophils, which produce IL-4 in mouse models of protease allergen-induced airway inflammation [74]. In humans, mast cells can promote ILC2 responses directly by producing prostaglandin D2 (PGD2) [75]. Furthermore, the epithelial cell-derived cytokine TGF-β plays a central role in enhancing ILC chemoactivity and generating the pulmonary immune response (Fig. 2) [67]. IL-33 is a chromatin-associated nuclear cytokine that is abundantly expressed in epithelial and endothelial cells and thought to be released only upon cellular damage or necrotic cell death [76, 77]. ILCs in the airway lumen respond to TGF-β through the receptor TGF-βRII [78]. IL-33 is over-expressed in the lungs of patients with idiopathic pulmonary fibrosis, asthma, and lung inflammation, and the over-expression of IL-33 is associated with ILC2 expansion [79]. IL-33 promotes ST2-dependent lung fibrosis by inducing macrophage activation and enhancing ILC2s expansion. Recent studies have shown that IL-33 is also expressed by NKT cells, alveolar macrophages, DCs, and mast cells [9, 80–82]. During influenza virus infection in mice, enhanced release of IL-33 from NKT cells occurred concomitantly with enhanced IL-5 production by ILC2s [82].

Moreover, T cells have been shown to interact with ILCs in the lung. ILCs were unable to induce worm expulsion in Rag2^−/−^ mice, which lack B and T cells, infected with helminths or challenged with the protease papain [83]. In mice lacking T cells, the number of ILC2s was reduced to the uninfected levels, despite the continuous presence of intestinal worms. These observations suggest that adaptive immune cells, such as T cells, promote ILC2 expansion and survival. Studies have also shown that ILC2s influence antigen-specific T cell responses. Adoptive transfer of wild type or Il13^−/−^ ILC2s into Il17br^−/−^ mice, in which the secretion of T-cell-derived IL-13 was delayed, restored the antigen-specific T cell production of IL-13. The results suggest that ILC2s regulate antigen-specific T cell responses and enhance T cell cytokine production in an IL-13-independent manner [83]. *In vitro* co-culture of lung ILC2s with CD4^+^ T cells induced the enhanced proliferation of the CD4^+^ T cells and the production of Th2 cytokines [83]. In papain-induced lung inflammation, IL-9 was produced by ILCs, and the production of IL-9 is dependent on IL-2 produced by T cells and B cells [84]. Furthermore, by increasing T helper 2 cell (T_h_2) responses, ILC2s can promote chronic inflammation in mice. This occurs either by migration of activated DCs to the lung draining lymph node and subsequent T_h_2 cell priming in response to IL-13 [85] or by the direct interactions with CD4^+^ T cells in a major histocompatibility complex class II (MHCII)-dependent manner [86, 87].

Crosstalk between B cell and ILCs in the lung has also been reported. The fat-associated lymphoid clusters (FALC)-derived ILC2s proliferate in response to IL-2 and produce large amounts of T_h_2 cytokines, including IL-5, IL-6 and IL-13. IL-5 and IL-6 regulate B cell antibody production and self-renewal of B1 cells [88–90]. Other studies showed that FALC-derived ILC2s support the self-renewal and expansion of B1 and B2 cells and enhance the production of the IgA, IgM, IgG1, and IgE antibody classes [34, 71, 72, 91]. However, a discrepancy was noticed in the results from different studies; therefore, further studies are needed to clarify the relationship between B cells and ILCs.

Studies have shown that IL-27 and IFN-γ, which can be released by ILC1s, antagonize the function of ILC2s and type 2 innate immune responses; in ILC2s lacking the IFN-γ receptor, ILC2-mediated lung inflammation was enhanced. The transcription factor STAT1 seems important in mediating the suppressive effects of IL-27 and IFN-γ on ILC2 functions [92, 93]. However, some other studies have shown that type I interferons directly and negatively regulate ILC2s in mice and humans by activating the transcription factor ISGF3 and the subsequent cytokine production, cell proliferation, and cell death [92]. Furthermore, although ILC3s are normally absent in the lungs of healthy mice [8], in the lungs of a mouse model of obesity-induced asthma, ILC3s expand in response to NLRP3-dependent production of IL-1β by macrophages [63]. Nonetheless, these findings suggest an interaction between ILC1s and ILC2s.

### ILCs mediate lung tissue repair

The recovery of lung tissue following injury is critical for restoring lung homeostasis and is a complex process involving multiple cellular and molecular regulators, such as interleukins (IL-1β, IL-2, IL-4, IL-9, and IL-13), chemokines (MCP-1), growth factors (TGF-β, KGF, and HGF), and extracellular matrix proteins (MMP-1, MMP-7, and MMP-9) [94–96]. Tissue remodeling following acute injury requires a balanced regulation between acute inflammation, the recruitment of immune cells, and epithelial cell proliferation. Failure of either appropriate cell proliferation or limitations in these repair responses can induce the loss of lung function, impair tissue integrity, and induce chronic inflammation or tissue fibrosis [94, 95]. It was found that the lung ILC population was critical for the repair and remodeling of damaged tissue following influenza virus infection [8]. Genome-wide transcriptional profiling revealed that lung ILCs express a number of genes associated with wound healing and tissue repair, including the extracellular matrix proteins decorin, aspirin and dermatopontin and epidermal growth factor family members, such as amphiregulin. Depletion of ILC2s did not impair innate immunity in the mice following influenza infection, but it did result in the loss of airway epithelial integrity, decreased lung function, and impaired airway remodeling [8]. This repair function was restored by administration of amphiregulin, the product of lung ILCs. In the study of *N. brasiliensis* infection in mouse lungs, autocrine IL-9 production contributes to the survival of activated ILC2s, amplifies ILC2-mediated amphiregulin production, and promotes tissue repair [96]. Therefore, ILC2s represent a major ILC population in the lung, which promotes the recovery of damaged lung tissue after infection.

### The role of ILCs in lung diseases

Current studies have recently emphasized the complex role of ILCs and their alterations in disease-association studies of patients and experimental models, such as lung transplantation, influenza virus infection, allergic asthma, chronic rhinosinusitis and chronic obstructive pulmonary disease (COPD).

In influenza virus infection, a population of ILCs that express CD90, CD25, CD127, and T1-ST2 was identified in the mouse and human lungs [8]. A mouse study showed that ILCs accumulated in the lung in response to IL-33 and immune-mediated tissue damage after influenza virus infection [8].

Asthma is a prevalent disease of chronic inflammation in which the Th2 cytokines IL-4, IL-5, and IL-13 are all tightly linked to the pathogenesis of asthma. IL-4 induces IgE production by B cells [97], IL-5 activates eosinophils and recruits them to the lung [98], and IL-13 increases mucus production [98]. Murine studies have shown that ILC2s accumulated in the lung and significantly contributed to IL-5 and IL-13 production in allergic asthma [99]. ILCs, including NK cells and ILC2s, participate in the regulation of allergic airway responses. NK cells were highly activated in patients with severe asthma and interacted with autologous eosinophils to promote their apoptosis. Both NK cells and ILC2s can decrease airway inflammation and mediate eosinophilic inflammation [75]. However, not all the functions of ILC2s are protective, as these cells also mediate pathological injury in a mouse model of virus-mediated exacerbation of allergic asthma. In animal models of allergic asthma, ILC2-derived IL-13 is an essential inducer of mucus hypersecretion and inflammation [100, 101]. IL-13 also causes inflammatory responses in monocytes and eosinophils, mucus cell metaplasia, airway fibrosis, and airway obstruction [102].

Chronic rhinosinusitis is a typical type 2 inflammatory disease associated with IL-13 release from ILC2s. Human ILC2s express a prostaglandin D2 receptor named chemoattractant receptor expressed on Th2 cells (CRTH2), and elevated numbers of CRTH2^+^ ILCs were observed in the nasal polyps of patients with chronic rhinosinusitis compared to control subjects [35, 77]. These cells responded to IL-2, IL-25 and IL-33, and their function depends on IL-13 [35]. Furthermore, anti-interleukin-13 therapy improves the lung function of patients with severe asthma who had a pretreatment profile consistent with interleukin-13 activity [103].

COPD is characterized by a chronic inflammation of the airways. The formation of lymphoid follicles and neutrophilic airway inflammation are two characteristics of COPD [104, 105]. In a mouse study, ILC accumulation in lung tissue was observed during acute exacerbations of COPD (AECOPD) [106]. A tendency toward a higher frequency of NCR^-^ ILC3 was observed in the lungs of patients with COPD compared with the controls [62]. Additionally, IL-17A and IL-22, which are produced by NCR^-^ ILC3s, contribute to the formation of lymphoid follicles [107, 108]. Neutrophil elastase and IL-5 levels are increased in AECOPD patients [109]. IL-13 mRNA levels are increased ~30-fold in sputum eosinophils and endothelial cells in AECOPD patients compared to normal control subjects [110]. Importantly, IL-5 and IL-13 were produced by ILC2s and are responsible for COPD exacerbation [111]. Although there is no study that reported the functions of ILCs in COPD, it is strongly tempting to speculate that ILC2s and ILC3s are activated in COPD patients.

### Potential therapeutic modulation of ILCs

Based on the findings showing that ILCs play an important role in lung homeostasis and diseases, there is an urgent need to investigate whether the modulation of ILC responses is a practical therapeutic strategy that could provide a clinical benefit. Indeed, various therapeutic strategies have been developed that aim to modify ILC development, cell-cell crosstalk, effector molecules, cytokine-receptor pathways, and tissue repair functions and maintenance. On the other hand, ILCs can be a biomarker for several diseases. Because many therapies were used to treat patients with multiple sclerosis or Crohn's disease, options are available for use to determine whether these strategies also work in patients with lung disease. A therapeutic example is the treatment of patients with multiple sclerosis with daclizumab, which is a humanized monoclonal antibody against α chain of the interleukin-2 receptor (IL-2Ra; CD25). Daclizumab modulates ILC responses by decreasing the number of ILCs by modifying the cells’ phenotypes and shifting the cells toward a NK cell lineage [112].

However, it is important to consider how the therapeutic strategies may affect the pathologic versus protective functions of ILCs. Some methods include targeting molecules that are critical for the development and migration of ILCs, e.g. α4β7 and MAdCAM-1. A clinical study revealed that Vitamin D can inhibit cytokine production and integrin α4β7 expression in human ILCs [113]. However, retinoic acid has antagonistic effects, which worked synergistically with other cytokines to induce the expression of the gut-homing integrin α4β7 in ILCs. Therefore, the balance between Vitamins A and D influences human ILC responses and acts as an critical factor in the function of ILCs in diseases, such as allergic inflammation [113]. Furthermore, some strategies target the effector molecules of ILCs, such as TNF-TNFR, IL-17-IL-17R, IFN-γ and IL-13-IL-13R. Many anti-Interleukin-17 or anti-Interleukin-23 receptor antibodies, which target the IL-23-IL-17 pathway, have beneficial effects on psoriasis and rheumatoid arthritis in experimental mouse models and patients [114–119]. However, in patients with Crohn's disease, the blockade of IL-17 was ineffective and caused higher rates of adverse events. In some cases, anti-IL-17 therapy resulted in increased susceptibility to fungal infections and enhanced disease [120–123]. Because ILC2s can regulate eosinophilic inflammation, a recent study showed that ILC2s acted as a surrogate biomarker of eosinophilic airway inflammation in patients. Moreover, this biomarker may distinguish the asthmatic patients with mild to moderate asthma who are most likely to receive benefits from therapeutics targeting Th2 inflammation [124]. Therefore, novel therapeutics are necessary for selectively modulating protective versus pathologic ILCs responses, including novel small-molecule inhibitors of transcription factors.

Despite these advances, further studies are required to reveal the ILC responses in defined patient populations and to fully reveal how ILC function can be modulated to limit human disease.

## Conclusions

Our knowledge of ILC development and regulation and their roles in lung diseases has been greatly advanced by recent research. However, a better definition of innate immune cells, i.e., a universal consensus on the markers for ILCs in humans and mice, is needed. At the cellular and molecular level, the interactions between ILCs and other immune and tissue cells, as well as the mechanisms involved in these interactions, need to be addressed. A critical appraisal of translational studies is also expected, including the determination of the expression profiles of these cells in patients and the ILC responses to currently used medications. Furthermore, several questions regarding the potential plasticity of ILC populations, ILCs’ novel functions, and the regulatory pathways affecting ILC responses should be addressed using animal approaches. Knowledge of ILC biology and their roles in resting conditions and disease states will be necessary for us to better understand the mechanisms of lung diseases and to develop novel therapeutic options for these diseases.

## Abbreviations

Ahr, Aryl hydrocarbon receptor; CHILP, Common helper innate lymphoid precursor; CLPs, Common lymphoid progenitors; COPD, Chronic obstructive pulmonary disease; CRTH2, Chemoattractant receptor expressed on Th2 cells; Eomes, Eomesodermin; FALC, Fat-associated lymphoid clusters; GATA3, GATA binding protein 3; HPCs, Hematopoietic progenitor cells; Id2, Inhibitor of DNA binding 2; IFN-γ, Interferon-γ; ILC1s, Group 1 ILCs; ILC2s, Group 2 ILCs; ILC3s, Group 3 ILCs; ILCps, ILC progenitors; ILCs, Innate lymphoid cells; IRF4, Interferon regulatory factor 4; LP, Lamina propria; LT, Lymphotoxin; LTi, Lymphoid tissue-inducer cells; NCR, Natural cytotoxicity receptors; NFIL3, Nuclear factor interleukin-3; NKps, NK cell precursors; NKs, Natural killer cells; PGD2, Prostaglandin D2; PLZF, Promyelocytic leukemia zinc finger; RAG, Recombination activating gene; ROR-α, Retinoic acid receptor-related orphan receptor-α; ROR-γt, Retinoic acid receptor-related orphan receptor-γt; SCA-1, Stem cell antigen 1; SCF, Stem cell factor; TCF1, T cell factor 1; Th2, T helper 2 cell; TNF, Tumor necrosis factor; TOX, Thymocyte selection–associated high-mobility group box protein; TSLP, Thymic stromal lymphopoietin
